# The effects of anticipation of standing surface translations on distal leg muscle excitations

**DOI:** 10.1007/s00221-025-07075-y

**Published:** 2025-04-15

**Authors:** Virginie Ruest, Emily Eichenlaub, Jason R. Franz

**Affiliations:** 1 Joint Department of Biomedical Engineering, University of North Carolina at Chapel Hill and North Carolina State University, Chapel Hill, NC USA; 2 10206C Mary Ellen Jones Building CB 7575, Chapel Hill, NC 27599 USA

**Keywords:** Falls, Neuromechanics, Posture, Anticipatory postural adjustments, Reactive postural adjustments

## Abstract

This study aimed to investigate the interactions between anticipation and direction of surface translations applied during standing on distal leg muscle excitations measured via surface electromyography (EMG) in the context of resultant changes in center of mass (CoM) displacements. Twenty young adults experienced four combinations of surface translations (i.e., anticipated anterior, unanticipated anterior, anticipated posterior, unanticipated posterior) via a dual-belt instrumented treadmill. For each condition, a 200 ms, 6 m/s^2^ anterior or posterior perturbation was delivered either unexpectedly or after a three-second verbal countdown. Each condition was repeated three times in a randomized order. We recorded surface EMG from the MG, SOL, and TA, and motion capture collected pelvis displacements as a surrogate for CoM. Data were analyzed across three time periods: pre-perturbation, early post-perturbation, and late post-perturbation. Direction-dependent EMG responses were as hypothesized, with TA iEMG increasing by 81% following anterior perturbations and MG and SOL iEMG increasing by 60% and 32% respectively following posterior perturbations. As hypothesized, anticipated balance challenges elicited greater proactive iEMG for all muscles. However, only unanticipated anterior perturbations elicited greater reactive TA iEMG than anticipated anterior perturbations. These proactive neuromechanical adjustments, particularly for anterior surface translations that would precipitate a backward fall, appeared protective and effective based on resultant patterns of CoM displacements. These data serve as a reference for understanding how aging and disease impact proactive and reactive postural control, especially for populations who may have difficulty with both the planning and execution of corrective neuromuscular adjustments.

**Supplementary Information:**

The online version contains supplementary material available at 10.1007/s00221-025-07075-y.

## Introduction

Balance integrity during standing is an important component to mitigating instability and minimizing the risk of falls (Dominguez 2020). With the number of older adults expected to increase from 58 million in 2022 to 82 million in 2050, there is an urgent need for continued study into mechanisms of instability and potentially modifiable factors for fall prevention (Mather and Scommegna 2024). The timing and amplitude of local muscle excitations can provide insight into how the neuromechanical system governs standing balance. Postural control arises as a complex interplay between proactive adjustments in anticipation of instability and/or reactive adjustments to recover from that instability (Xie and Wang 2019). Balance perturbations, whether anticipated or unanticipated, allow us to quantify changes in local muscle neuromechanics that, in younger adults, can serve as a benchmark for detrimental changes due to aging and disease.

Surface translations are a context of standing balance perturbations that can facilitate measurements of postural corrections used to maintain stability. For example, we know that muscle excitations deployed to counteract a backward loss of balance (i.e., anterior translations) differ from those deployed to counteract a forward loss of balance (i.e., posterior translations) (Bhatt et al. 2006, 2013; Wang et al. 2012; Eichenlaub et al. 2023). A stretch reflex caused by joint rotation elicits a rapid muscle force response, typically measured via an increase in agonist electromyography (EMG). The opposing muscle is inhibited through reciprocal Ia pathways (Tang et al. 1998; Martino et al. 2023). For example, an unanticipated posterior surface translation precipitates rapid tibialis anterior (TA) shortening with medial gastrocnemius (MG) and soleus (SOL) lengthening (Henry et al. 1998). To prevent a forward fall, the ankle joint engages in plantarflexion by contracting the plantarflexor muscles (e.g., the MG and SOL). Conversely, an anterior surface translation precipitates rapid MG and SOL shortening with TA lengthening (Henry et al. 1998). To prevent a backward fall, the ankle joint engages in dorsiflexion by contracting the dorsiflexor muscles (e.g., the TA). Considering their biomechanical roles, we anticipate that the TA will exhibit higher EMG activity in response to anterior surface translations while the MG and SOL will show increased EMG activity following posterior surface translations.

Compared to the reactive corrective motor responses discussed above, anticipation of a balance perturbation intuitively precipitates an anticipatory feedforward motor response to prepare for pending instability (Bastian 2006; Duarte et al. 2022). Much like the reactive postural adjustments (RPAs) following perturbation onset, these anticipatory postural adjustments (APAs) can also be quantified via surface EMG (Horak 2006; Smith and Fisher 2018; Duarte et al. 2022). There is significant potential for interplay between APAs and RPAs. APAs can be deployed to prepare the body for an anticipated perturbation and thereby reduce the demand for RPAs, and thereby the potentially substantial demands on muscle force and power generation (Marigold and Patla 2002; Pavol et al. 2004; Bastian 2006; Chambers and Cham 2007; Chvatal and Ting 2012; Eichenlaub et al. 2023). Conversely, effective reactive responses to unanticipated perturbations rely on the early onset of stretch reflexes that initiate muscle contractions to counteract the disturbance, leading to a rapid increase in muscle activation to regain stability (Tang et al. 1998; Marigold and Patla 2002). Agonist muscles also exhibit direction-dependent behaviors when preparing for and reacting to a balance challenge, which suggests that the body employs distinct strategies for balance control and recovery from instability (Eichenlaub et al. 2025). However, the combined effects of anticipation and perturbation direction on lower-limb muscle excitations remain unexplored. In this study, we employ anticipation as a framework to investigate the neuromechanical changes in muscle activation involved in both proactive and reactive balance control.

The purpose of this study was to investigate the interactions between anticipation and direction on distal leg muscle excitations measured via surface electromyography (EMG) before and after standing surface translations. We hypothesized that unanticipated balance challenges would elicit greater reactive EMG following a perturbation while anticipated balance challenges would elicit greater proactive EMG preceding a perturbation. Finally, we interpreted these outcomes in the context of CoM displacement patterns measured via anterior-posterior pelvis kinematics.

## Materials and methods

### Participants

20 young adults (8 males/12 females; mean ± standard deviation; age: 22.3 ± 3.3 years; height: 1.72 ± 0.13 m; mass: 65.8 ± 12.3 kg) participated in this single-visit study approved by the University of North Carolina Biomedical Sciences Institutional Review Board. Written informed consent was obtained from each participant prior to data collection. All participants could walk without an assistive device and confirmed absence of neurological disorders and lower extremity injuries or fractures within the last six months.

### Experimental protocol and data collection

**Fig. 1:**
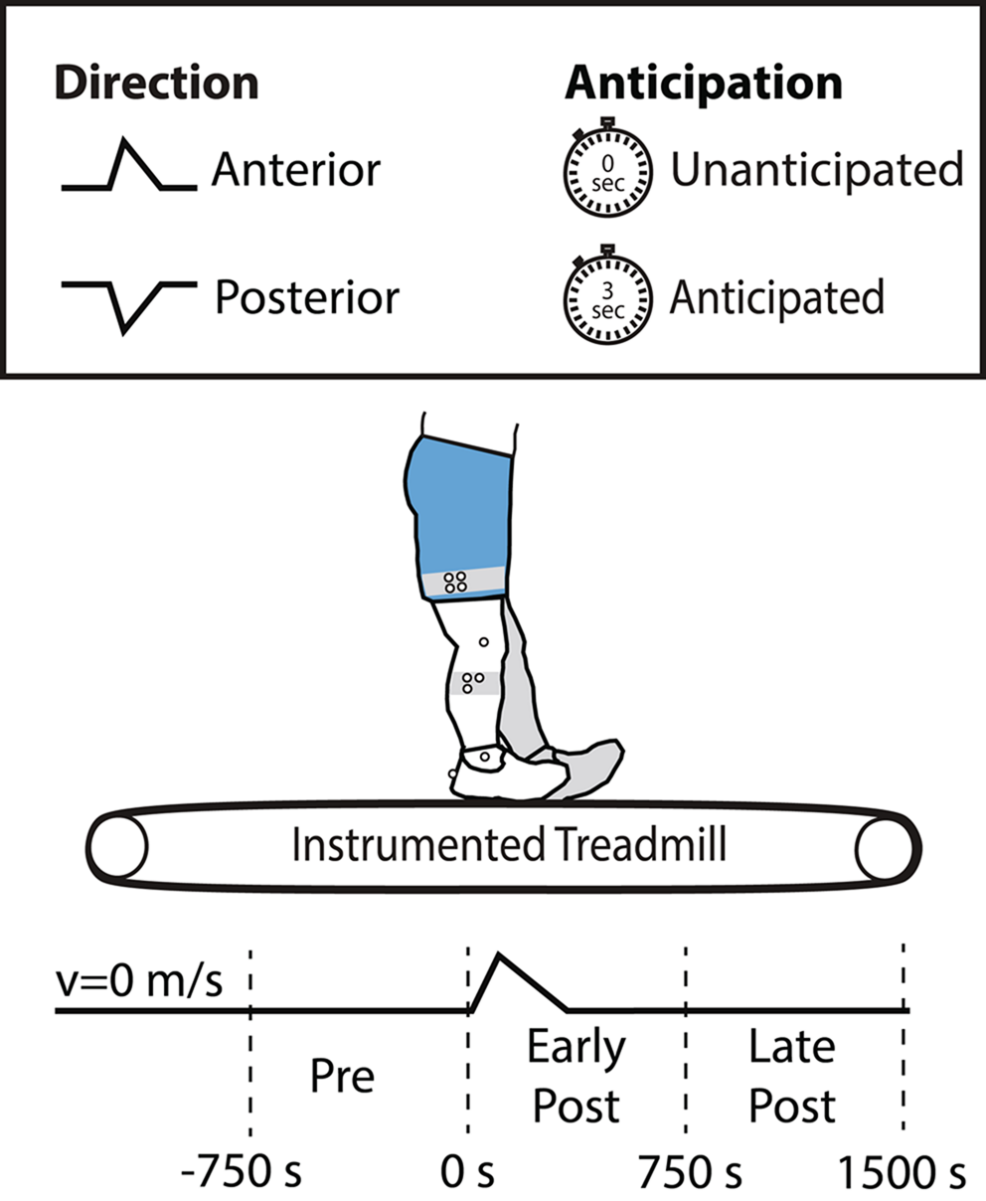
Four combinations of surface translations (anticipated and unanticipated, anterior and posterior) were applied during standing via a dual-belt instrumented treadmill. Participant responses were assessed across three time periods: pre-perturbation (750 ms before onset), early post-perturbation (0–750 ms), and late post-perturbation (750–1500 ms)

Figure 1 summarizes our experimental protocol. Each experimental trial consisted of a 200 ms, 6 m/s^2^ anterior or posterior treadmill surface translation applied simultaneously to both legs via a dual-belt instrumented treadmill (Bertec, Inc., Columbus, OH). We designed these treadmill-belt perturbations to replicate those used in prior studies (Lee et al. 2016; Liu et al. 2016; Eichenlaub et al. 2023). Participants were instructed to stand with their feet positioned at a comfortable, natural distance from each other. This distance was measured and consistently maintained throughout the experiment. Participants were asked to avoid taking steps during the task, although stepping was permitted if necessary for stability. Trials in which participants took a step were omitted from analysis. Perturbations were delivered either unexpectedly (i.e., unanticipated) or at the end of a three-second verbal countdown (i.e., anticipated) using a custom MATLAB interface (MathWorks, Natick, MA, USA). To ensure participants could not predict the timing of the unanticipated perturbations, the researcher delivered perturbations at random intervals and the participant’s foot placement was reset before each trial. Four combinations of perturbation direction (i.e., anterior or posterior) and anticipation (i.e., anticipated or unanticipated) were repeated three times in a randomized order, both within and among participants, for a total of 12 perturbations. We recorded electromyographic (EMG) signals from the left MG, SOL, and TA during each trial using wireless surface electrodes operating at 1000 Hz (Delsys, Natick, MA, USA). Electrodes were placed over the respective muscle bellies according to SENIAM guidelines (seniam.org). Simultaneously, a 15-camera motion capture system (Motion Analysis Corporation, Santa Rosa, California, USA) operating at 100 Hz captured the 3D trajectory of a marker placed on the posterior sacrum as a surrogate for the body’s center of mass (CoM).

### Data analysis

Each muscle’s raw EMG signal was demeaned, filtered using a 4th order bandpass (20–400 Hz) Butterworth filter, and full wave rectified. We then obtained 10-Hz linear envelopes using a lowpass filter and calculated integrated EMG (iEMG) over three distinct time periods: (i) pre-perturbation (i.e., 750 ms preceding perturbation onset), (ii) early post-perturbation (i.e., 0-750 ms following perturbation onset), and (iii) late post-perturbation (i.e., 750–1500 ms following perturbation onset) (Martino et al. 2023). Anterior-posterior CoM displacements were low-pass filtered at 16 Hz, averaged across trials, and analyzed over the same three distinct time periods. Specifically, the primary outcome was the range of CoM displacement exhibited during a respective epoch.

### Statistical analysis

Anterior-posterior CoM displacements and EMG outcomes from the MG, SOL, and TA were analyzed separately within each of the three time periods (i.e., pre-perturbation, early post-perturbation, and late post-perturbation) and were averaged across the three trial repetitions per condition. We determined main effects of and interactions between direction and anticipation using an aligned ranks transformation (ART) analysis of variance (ANOVA). A significance level of *p* ≤ 0.05 was used for all comparisons.

## Results

**Fig. 2:**
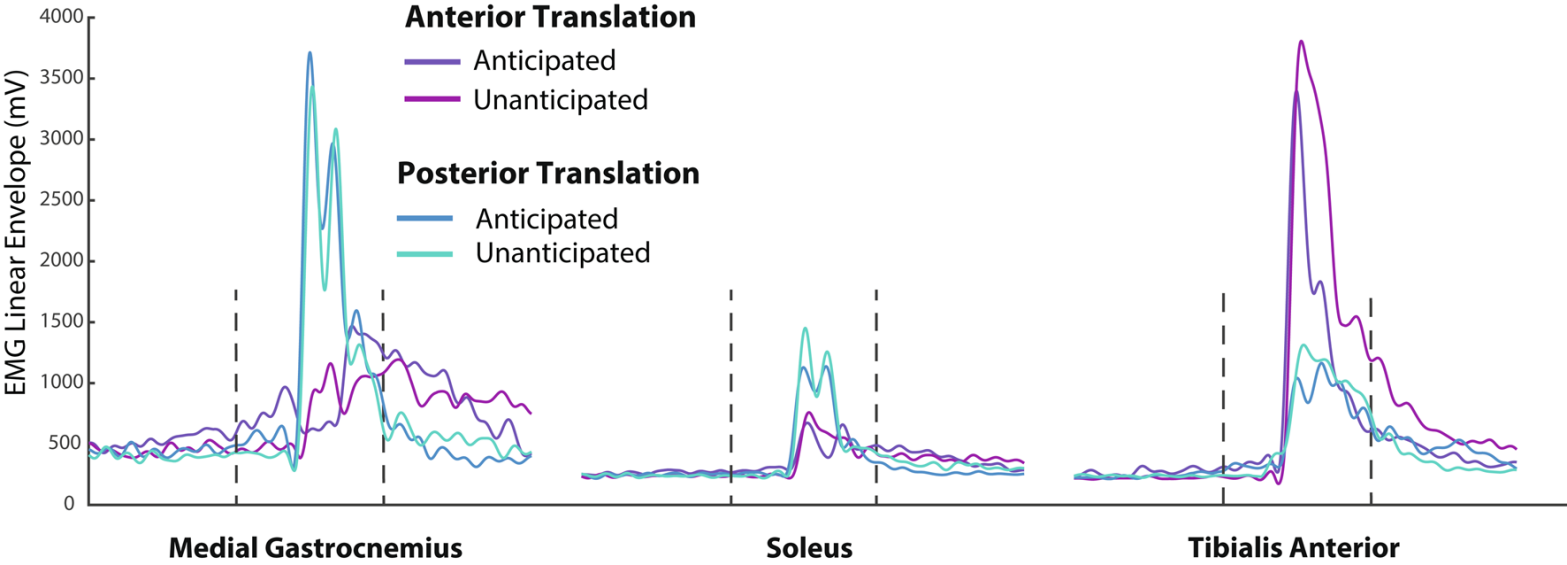
EMG linear envelopes for the medial gastrocnemius (MG), soleus (SOL), and tibialis anterior (TA) in response to treadmill-belt perturbations beginning 750 ms prior to perturbation onset through 1500 ms after perturbation onset. Vertical dashed lines indicate perturbation onset (0 ms, left) and the end of the early post-perturbation epoch (+ 750 ms, right)

**Fig. 3:**
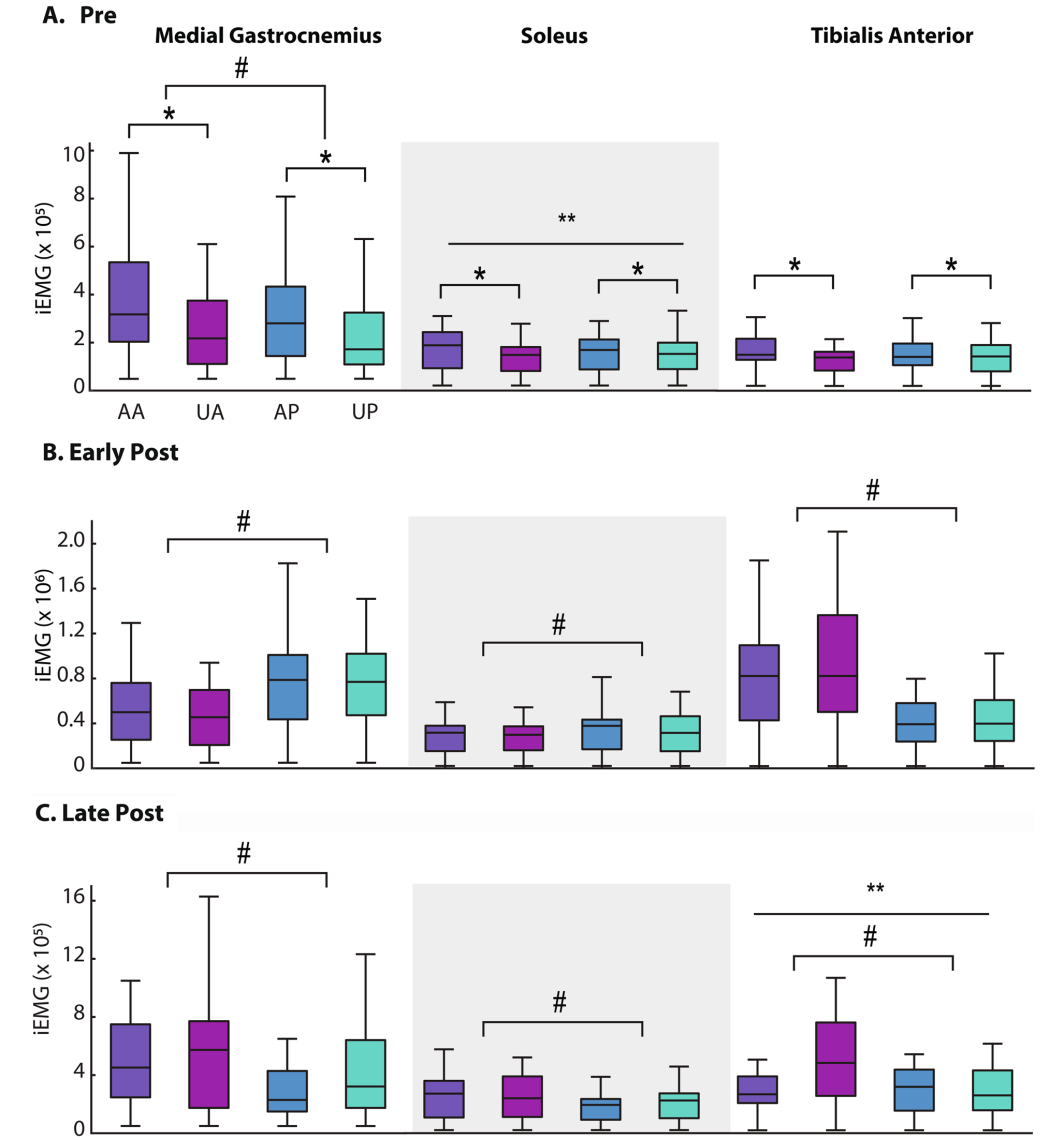
Integrated EMG (iEMG) for the medial gastrocnemius (MG), soleus (SOL), and tibialis anterior (TA) during the pre-perturbation (-750 ms to 0 ms, A), early post-perturbation (0 ms to 750 ms, B), and late post-perturbation (750 ms to 1500 ms, C). AA: Anticipated Anterior; UA: Unanticipated Anterior; AP: Anticipated Posterior; UP: Unanticipated Posterior. Single asterisks (*) indicate significant pairwise effects of anticipation, hashtags (#) indicate significant effects of direction, and double asterisks (**) indicate significant interactions between anticipation and direction

**Fig. 4:**
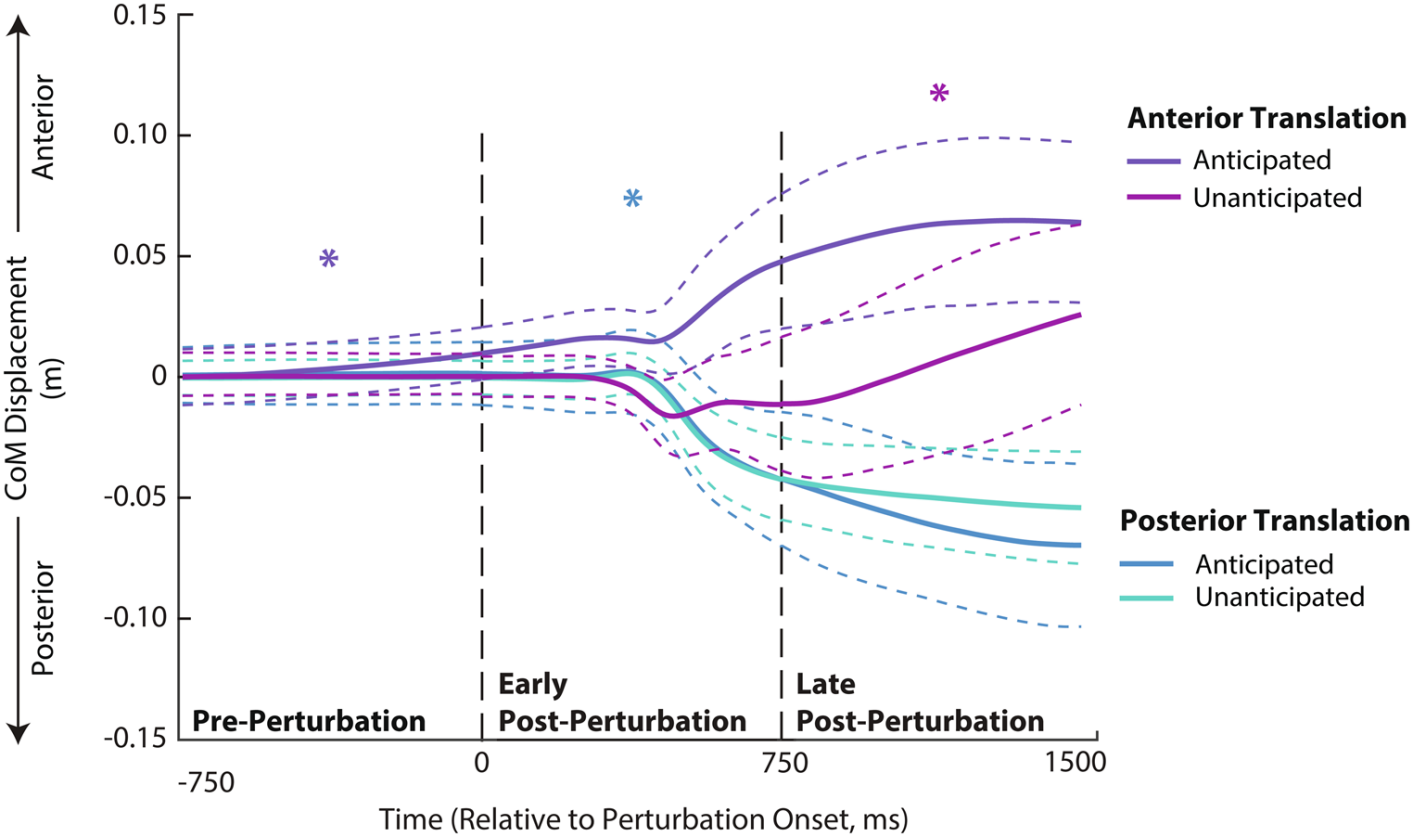
Center of mass (CoM) displacements estimated via anterior-posterior sacrum position in response to treadmill-belt perturbations. Significant pairwise effects of anticipation are shown by colored single asterisks (*), wherein the color refers to the condition with larger displacement

Figure 2 shows linear envelopes of MG, SOL, and TA EMG profiles, which are quantified via integrated EMG (iEMG) shown in Fig. 3. Finally, Fig. 4 shows the resultant patterns of CoM displacements.

### Pre-perturbation

We found a significant main effect of direction on MG iEMG (*p* = 0.003) wherein, independent of anticipation, anterior perturbations elicited greater MG iEMG than posterior perturbations with a difference of 16% (Fig. 3a). Here also, independent of direction, anticipation increased MG iEMG (*p* < 0.001) by 15%, SOL iEMG (*p* < 0.001) by 8%, TA iEMG (*p* = 0.039) by 11%, and CoM displacement (*p* < 0.001, Fig. 4) by 100%. We also found an anticipation×direction interaction (*p* = 0.02) for SOL iEMG. Here, anticipated anterior perturbations elicited greater SOL iEMG than all other conditions by 8–12%.

### Early post-perturbation

We found a main effect of perturbation direction on MG (*p* < 0.001), SOL (*p* = 0.001), and TA (*p* < 0.001) iEMG (Fig. 3b). Specifically, posterior perturbations elicited greater reactive MG and SOL iEMG with differences of 60% and 32% respectively, whereas anterior perturbations elicited greater reactive TA iEMG with a difference of 81%. We also found significant main effects of anticipation (*p* = 0.012), direction (*p* < 0.001), and an anticipation×direction interaction (*p* = 0.031) for CoM displacement (Fig. 4). Here, anticipated posterior translations were accompanied by larger CoM displacements with a difference of 4–58%.

### Late post-perturbation

We found significant main effects of direction on MG (*p* < 0.001), SOL (*p* < 0.001), and TA (*p* = 0.002) iEMG wherein, independent of anticipation, anterior perturbations elicited greater reactive iEMG than posterior perturbations with differences of 83%, 28%, and 31%, respectively (Fig. 3c). We found a significant main effect of anticipation (*p* = 0.004) on CoM displacement, wherein unanticipated perturbations elicited larger CoM displacement than anticipated perturbations by 16%. We also found a significant main effect of direction (*p* = 0.003) on CoM displacement, wherein anterior perturbations elicited greater CoM displacement than posterior perturbations by 23%. Lastly, significant anticipation×direction interaction effects for TA iEMG (*p* < 0.001) and CoM displacement (*p* = 0.002) revealed that unanticipated anterior perturbations elicited 42–82% greater reactive iEMG and 17–59% larger CoM displacements compared to all other conditions (Fig. 4).

## Discussion

This study aimed to investigate the interactions between anticipation and direction of surface translations applied during standing on distal leg muscle excitations measured via surface electromyography in the context of resultant changes in CoM displacements. Taken together, our results provide compelling support for our hypothesis. For anterior perturbations, the TA acts as the primary agonist, whereas the MG and SOL take on this role for posterior perturbations. Accordingly, the TA showed greater EMG following anterior surface translations while the MG and SOL showed greater EMG following posterior perturbations. Moreover, our results support the interpretation that anticipated balance challenges elicited greater proactive EMG, evidenced by greater MG, SOL, and TA iEMG pre-perturbation. Simultaneously, these neuromechanical adjustments, particularly for anterior surface translations that would precipitate a backward fall, appeared protective and effective based on resultant patterns of CoM displacements. Specifically, anticipation led to increased CoM displacements pre-perturbation, reflecting anticipatory postural adjustments. Anticipation of anterior translations was accompanied by larger CoM displacements early post-perturbation, likely playing a protective role in preventing a backward fall. We also found one example that supported our hypothesis that unanticipated perturbations would elicit greater reactive EMG; specifically, during the late-post perturbation period, unanticipated anterior translations elicited greater TA iEMG and larger CoM displacements than when anticipated. Though not something that generalized to posterior translations, those results imply that additional corrective strategies such as RPAs may be necessary to mitigate instability. These data can now be used as a reference for understanding how aging and disease impact proactive and reactive postural control, especially for populations who may have difficulty with both the planning and execution of corrective neuromuscular adjustments.

In agreement with existing literature and our hypothesis, participants employed proactive control strategies to maintain their balance in preparation for anticipated perturbations via increased agonist activation (Chambers and Cham 2007). Regardless of direction, anticipation led to higher MG, SOL, and TA proactive activation in comparison to the unanticipated condition, suggesting that the nervous system increased muscle activity before the perturbation occurred. For the riskier anterior surface translations, those changes precipitated or were at least accompanied by protective anterior displacements of the body’s CoM– an opportunity to mitigate the pending and anticipated posterior displacements. Higher activity likely reflects a strategy of antagonist coactivation, which serves to stiffen the body in preparation for pending perturbations (Milner et al. 1995; Finley et al. 2012; Thompson et al. 2018). By engaging both agonist and antagonist muscles, participants can better stabilize their posture, a tactic that may be especially relevant for individuals who are more fearful of instability. Such proactive adjustments can reduce the necessity for reactive adjustments (Marigold and Patla 2002; Pavol et al. 2004; Bastian 2006; Chambers and Cham 2007; Chvatal and Ting 2012; Eichenlaub et al. 2023).

Immediately after a perturbation, the body relies on localized stretch reflexes to maintain stability and prevent falls. According to Henry et al., posterior perturbations cause an initial stretch reflex in the MG and SOL while anterior perturbations elicit an initial stretch reflex in the TA (1998). Here, we found direction-specific reactive muscle activations, as only the TA exhibited greater reactive activation in response to unanticipated anterior surface translations during the last 750-ms epoch. Prior work has shown that when confronted with unanticipated perturbations, participants do not adjust their posture to counteract the effects of a pending disturbance, likely requiring larger reactive neuromuscular corrections (Horak 2006; Smith and Fisher 2018; Duarte et al. 2022). Our results are consistent with this premise. Moreover, we find evidence that the proactive neuromuscular adjustments deployed in anticipation of anterior surface translations were protective; proactive anterior CoM shifts prior to perturbation onset prevented the larger CoM displacement evident when those perturbations were unanticipated.

While early reactive responses rely on short latency reflexes, late reactive adjustments involve more complex neural pathways that integrate sensory feedback and higher-level processing (Christensen et al. 2000; Morton and Bastian 2004; Bastian 2006; Finley et al. 2012). Over time, the body integrates ongoing sensory feedback to adjust its response, allowing for more refined motor control (Finley et al. 2012). The cerebellum plays a crucial role in this process by processing sensory information and projecting to the motor cortical areas, influencing feedforward control and motor planning (Morton and Bastian 2004; Bastian 2006). During the late post-perturbation period (i.e., 750–1500 ms), unanticipated anterior translations led to increased activation of the agonist muscle (i.e., TA), further emphasizing that reactive responses are intensified in the absence of APAs. Notably, young adults responded to anterior perturbations (i.e., those that would compel a backward fall) by increasing activation of their plantarflexor muscles (i.e., MG and SOL). This response may require greater SOL-TA muscle coactivation to increase ankle joint stiffness, as this has been shown to be a strategy to maintain postural stability (Milner et al. 1995; Finley et al. 2012; Thompson et al. 2018). Finally, accompanying CoM displacements during the late post-perturbation period continue to demonstrate the efficacy of the proactive neuromuscular adjustments deployed by younger adults when perturbations were anticipated. Specifically, muscle actions that induced or at least were accompanied by protective anterior CoM displacements prior to perturbation onset ultimately lessened the resultant displacements observed during the late post-perturbation period. Thus, the patterns of proactive adjustments deployed by our healthy younger adults provide a benchmark for protective and effective strategies to mitigate instability.

To gain a better understanding of how anticipation and perturbation direction influence responses to surface translations and thus inform strategies to mitigate instability and minimize the risk of falls, we suggest reproducing this study in older adults as well as in individuals affected by neurological conditions, such as those with cognitive decline. Age-related changes in postural control, which contribute significantly to fall risk, can be identified by comparing the proactive and reactive postural adjustments of older adults with those of younger adults (Smith and Fisher 2018; Duarte et al. 2022). We anticipate that older adults may exhibit less effective proactive adjustments and greater reliance on reactive strategies, potentially leading to increased instability. We hypothesize that, in healthy older adults, reactive balance will be impaired, and that older adults with cognitive decline will experience greater impairments in both proactive and reactive balance, likely due to compromised executive function. Additionally, we recommend incorporating ultrasound technology to assess muscle dynamics, as muscle excitation is not necessarily a surrogate for muscle force. This approach would allow for a more comprehensive evaluation of the mechanical state (i.e., length and velocity) of the muscles involved, providing deeper insights into how these factors contribute to balance and stability.

This study has several limitations. One limitation was that participants were always aware that they could be perturbed at any moment and thus we cannot exclude the potential for postural adjustments even for unanticipated perturbations. We randomized the delivery of anticipated and unanticipated perturbations, as well as their direction, to minimize this effect. The absence of CoM displacements during the pre-perturbation period for unanticipated perturbations should mitigate concerns of this limitation. In addition, we averaged three trials for each experimental condition. Repeated exposure may have an impact on muscle activations and patterns of CoM displacement (Wang et al. 2022; Eichenlaub et al. 2023). Quantifying adaptation to perturbation exposure was outside the scope of the present study. Though, we suspect that the effects of this methodological decision are likely to only lessen the magnitude of changes we would expect in the absence of any adaptation. Another limitation is that we averaged the absolute values of iEMG across males and females. However, a post-hoc exploratory analysis revealed no sex-related main effects across any experimental condition– an outcome that builds confidence in our methodological decision.

## Conclusion

Our results in younger adults provide a significant first step toward understanding the role of anticipation and perturbation direction in governing the neuromechanical integrity of distal leg muscles and the resultant changes in CoM dynamics in mitigating instability. Our findings now serve as an important benchmark for identifying detrimental changes due to aging or disease and thereby risk factors or potentially modifiable factors for intervention.

## Electronic supplementary material

Below is the link to the electronic supplementary material.

Supplementary Material 1

## Data Availability

Data is provided within the manuscript or supplementary information files.
